# Comparison of high protein and high fiber weight-loss diets in women with risk factors for the metabolic syndrome: a randomized trial

**DOI:** 10.1186/1475-2891-10-40

**Published:** 2011-04-28

**Authors:** Lisa A Te Morenga, Megan T Levers, Sheila M Williams, Rachel C Brown, Jim Mann

**Affiliations:** 1 Department of Human Nutrition, University of Otago, PO Box 56, Dunedin 9054, New Zealand; 2 Department of Preventive and Social Medicine, University of Otago, PO Box 56, Dunedin 9054, New Zealand; 3 Edgar National Centre for Diabetes Research, University of Otago, PO Box 56, Dunedin 9054, New Zealand; 4 Riddet Institute, Private Bag 11 222, Palmerston North 4442, New Zealand

## Abstract

**Background:**

Studies have suggested that moderately high protein diets may be more appropriate than conventional low-fat high carbohydrate diets for individuals at risk of developing the metabolic syndrome and type 2 diabetes. However in most such studies sources of dietary carbohydrate may not have been appropriate and protein intakes may have been excessively high. Thus, in a proof-of-concept study we compared two relatively low-fat weight loss diets - one high in protein and the other high in fiber-rich, minimally processed cereals and legumes - to determine whether a relatively high protein diet has the potential to confer greater benefits.

**Methods:**

Eighty-three overweight or obese women, 18-65 years, were randomized to either a moderately high protein (30% protein, 40% carbohydrate) diet (HP) or to a high fiber, relatively high carbohydrate (50% carbohydrate, > 35 g total dietary fiber, 20% protein) diet (HFib) for 8 weeks. Energy intakes were reduced by 2000 - 4000 kJ per day in order to achieve weight loss of between 0.5 and 1 kg per week.

**Results:**

Participants on both diets lost weight (HP: -4.5 kg [95% confidence interval (CI):-3.7, -5.4 kg] and HFib: -3.3 kg [95% CI: -4.2, -2.4 kg]), and reduced total body fat (HP: -4.0 kg [5% CI:-4.6, -3.4 kg] and HFib: -2.5 kg [95% CI: -3.5, -1.6 kg]), and waist circumference (HP: -5.4 cm [95% CI: -6.3, -4.5 cm] and HFib: -4.7 cm [95% CI: -5.8, -3.6 cm]), as well as total and LDL cholesterol, triglycerides, fasting plasma glucose and blood pressure. However participants on HP lost more body weight (-1.3 kg [95% CI: -2.5, -0.1 kg; p = 0.039]) and total body fat (-1.3 kg [95% CI: -2.4, -0.1; p = 0.029]). Diastolic blood pressure decreased more on HP (-3.7 mm Hg [95% CI: -6.2, -1.1; p = 0.005]).

**Conclusions:**

A realistic high protein weight-reducing diet was associated with greater fat loss and lower blood pressure when compared with a high carbohydrate, high fiber diet in high risk overweight and obese women.

## Background

Debate continues regarding the most appropriate macronutrient composition for achieving weight loss and improvement in risk factors for cardiovascular disease (CVD) and diabetes in overweight and obese individuals [1,2]. The "standard" low fat high carbohydrate (LFHC) approach, long recommended because of its cardio-protective potential, has been repeatedly challenged during the past decade by proponents of Mediterranean type diets [3], high fat diets [4-6] and more recently high protein diets (e.g. Zone [7], CSIRO Total Wellbeing diet [8]). Those advocating these alternatives to the LFHC approach have generally claimed greater weight reduction and or more favorable metabolic profiles [9]. Given that many who are overweight and obese have risk factors associated with the metabolic syndrome, the potential of high protein diets to reduce triglyceride concentrations and blood pressure while maintaining HDL cholesterol levels is regarded as being especially relevant [10].

The findings of trials that have compared high protein and LFHC diets have not been entirely consistent. Some, which have suggested a more favorable response to high protein have utilized LFHC comparison diets have included relatively high intakes of refined grains, starchy vegetables and sugars [11-14]. This is inappropriate since the benefits of high carbohydrate diets have only been observed when most dietary carbohydrate has been derived from fiber-rich wholegrain cereals, pulses, vegetables and fruit [15]. Other studies have used amounts of protein which would have been impractical for long term consumption [16,17] or have only demonstrated benefit in subgroups such as those with elevated triglyceride concentrations [18] or women [19]. Thus, in a "proof of concept" study, we have compared a diet high in protein (HP) with one high in fiber-rich, minimally processed cereals and legumes (HFib) to determine whether acceptable high protein diets have the potential to confer greater benefits.

## Methods

Women aged 18-65 years with a body mass index (BMI) ≥ 27 kg/m^2 ^wishing to lose weight were recruited via advertisement in a local newspaper. Potential participants were screened during a telephone interview to determine eligibility, commitment to a nutrition-focused weight-loss program and existence of exclusion criteria. Potential participants were excluded if heart disease, cancer or kidney disease had been diagnosed; if they were taking medications influencing appetite and weight control; if they had participated in a weight loss program or had lost more than 1 kg bodyweight in the previous two months; or if they were pregnant, planning a pregnancy or breastfeeding. On the basis of the telephone call eighty-seven participants were invited to attend a screening visit during which a questionnaire relating to personal, demographic and health details was completed. The study protocol, risks and benefits were explained to each subject and written consent was given. The study was approved by the University of Otago Human Ethics Committee.

**Figure 1:**
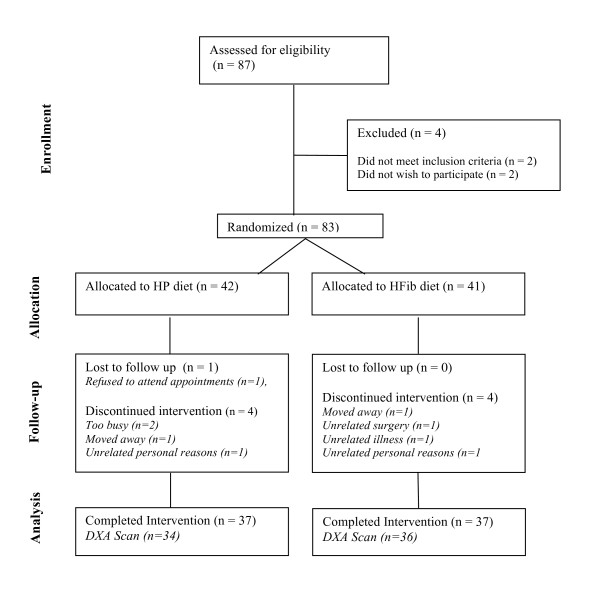
**Consort diagram showing flow of participants through the trial**.

Two women did not meet the inclusion criteria and two withdrew before being randomized, thus 83 women were randomly assigned to treatment. One participant withdrew early because of unrelated surgery, two moved away and six others (4 on HP and 2 on HFib) opted out of the intervention and were lost to follow-up. Seventy-four women (89% of those randomized) completed the entire study (Figure 1).

The study involved an 8-week randomised, controlled, partially blinded dietary intervention. Participants were randomly assigned to either a high protein (HP) or a high fiber, high carbohydrate (HFib) energy-restricted diet using sequentially numbered, sealed envelopes containing a computer-generated allocation using random length blocks and stratified by age and BMI. Laboratory staff and those conducting dual X-ray absorptiometry (DXA) scans were unaware of group allocation but participants and those involved in the dietary intervention or making clinical measurements could not be blinded regarding group allocation.

The HP diet was based on the CSIRO Total Wellbeing diet [8] in which approximately 30% of total energy (TE) is derived from protein, and 40% TE from carbohydrate. The HFib diet was designed to achieve at least 50% TE from carbohydrate, 20% TE from protein, and 35 g or more dietary fiber daily with emphasis on wholegrains and legumes. Total and saturated fat intakes were intended to be below 30% and 10% TE respectively. Energy intakes were designed to achieve weight loss of 0.5 - 1.0 kg per week requiring an energy deficit on both diets of approximately 2000-4000 kJ per day but with total energy of more than 5500 kJ per day to ensure adequate nutrient intakes.

Dietary instruction was centered on advice regarding the number of standard servings of key food groups to be eaten each day utilising exchange lists. The basic diet plan for the HP group recommended three 100 g servings of lean protein foods and three servings of breads, cereals or grains per day. The basic diet plan for the HFib group recommended one 100 g serving of lean protein foods, six servings of wholegrain breads, cereals or grains per day and one serving of legumes. Both diet groups received the same recommendations with regard to fruit, vegetable, and fat/oils servings (Table 1). The basic diet plans were individually tailored by adjusting the number of servings of major food groups to achieve the desired level of weight loss while maintaining the appropriate macronutrient composition, taking into account a participant's estimated basal metabolic rate and confidence to reduce energy intake. Participants were required to complete a daily food group checklist in order to maintain adherence to macronutrient and energy intake goals. The HFib group was also asked to estimate their daily fiber intakes using a simple fiber calculator and to aim for an intake of 40 g per day by selecting high-fiber breads, cereals, fruits, vegetables, nuts and legume choices. To encourage compliance to the dietary regime participants on the HFib diet were provided with six servings per day of key high carbohydrate foods including wholegrain bread, wholegrain cereal, bran cereal, rye crackers and canned legumes. The HP group was provided with grocery vouchers equivalent in value to the food items received by the HFib group. They were instructed to use the vouchers to purchase lean protein foods such as lean meat, fish and chicken and to keep their receipts as proof of purchase. Both groups were given material especially prepared for this study, including checklists, recipes and menu plans. Participants met with nutritionists at randomization and every 2 weeks throughout the study to encourage dietary adherence. At these sessions participants were weighed, daily food group checklists were reviewed and strategies for maintaining adherence to the dietary advice were discussed. Participants were asked to maintain their usual levels of exercise for the duration of the study.

**Table 1:** Food group recommendations for basic HP and HFib diet plans

HP Diet		HFib Diet	
**Food group**	**Servings per day**	**Food group**	**Servings per day**

		Beans and legumes (1/2 cup cooked)	1
Breads cereals & grains (approx 15 g carbohydrate)	3	High-fiber breads, cereals & grains (approx 15 g carbohydrate)	6
Lean protein foods (100 g raw weight)	3	Lean protein foods (100 g raw weight)	1
Low-fat milk and dairy foods (1 cup or equivalent kJ)	2	Low-fat milk and dairy foods (1 cup or equivalent kJ)	2
Vegetables (1/2 cup)	5	Vegetables (1/2 cup)	5
Fruit (medium size)	2	Fruit (medium size)	2
Fats and oils (teaspoon)	3	Fats and oils (teaspoon)	3
Indulgence foods and alcohol (approx 400 kJ)	2 per week	Indulgence foods and alcohol (approx 400 kJ)	2 per week

Participants completed a weighed 3-day diet record including 2 non-consecutive weekdays and one weekend day prior to commencing the intervention and at week 8 [20]. They were given instruction on keeping the diet record and were provided with electronic food scales. Dietary intakes of macronutrients and dietary fiber were calculated using the Diet Cruncher for Macintosh V1.2.0 program (Waydown South Software), which uses the New Zealand food composition database (Crop and Food New Zealand). At the end of each dietary data collection day, participants were asked to rate their hunger, fullness, thirst, pre-occupation with thoughts of food, desire to eat and how much they could have eaten over that day on a 10 cm visual analogue scale (VAS) [21].

To assess whether there was any change in physical activity level during the intervention participants completed the short International Physical Activity Questionnaire (Craig et al., 2003) at baseline and at week 8. At the final week 8 clinic visit participants completed an exit questionnaire where they were asked to rate their responses to a number of questions relating to the diet they followed on a scale of 0 - 10 where 0 was the extreme negative response and 10 was the extreme positive response.

Measurements were made on two occasions (to minimize intrapersonal variation) 2-7 days apart at baseline and at week 8 after a 10-hr overnight fast. Each participant's height, weight, waist circumference and resting blood pressure were measured in duplicate. Height was measured to the nearest millimeter using a stadiometer. Weight was measured in light clothing on electronic scales (Wedderburn) to the nearest 0.05 kg. Waist circumference (WC) was measured to the nearest millimeter using a standard non-stretching tape measure at the midpoint between the lowest part of the rib and highest part of the hip underneath clothing. Resting blood pressure was measured using a manual sphygmomanometer with participants in a seated position after resting for 5 minutes and then repeated 5 minutes later. A fasting blood sample was then taken for the measurement of lipids, glucose, insulin and high-sensitivity C-reactive protein (Hs-CRP).

Total body fat mass, lean mass, body fat percentage, and truncal fat mass were assessed by DXA (DPX-L scanner, Lunar Corp, Cincinnati, OH) using software version 1.35 (Lunar, Cincinnati, OH) at the Dunedin Public Hospital DXA Scanning Unit at baseline and week 8. DXA scanning was limited to participants weighing less than 120 kg (n = 70).

Whole blood samples were centrifuged at 1650 g for 15 minutes, then samples were pipetted into polyethylene cryovials and stored at -80°C. Laboratory results at all time-points for all subjects were performed in batch within the same assay. Serum insulin and C-peptide were measured using a specific insulin electrochemiluminescence immunoassay (ECLIA) (Roche, Cat. No. 12017547) for the Elecsys^® ^analyzer (Roche Diagnostics, Mannheim, Germany), with a coefficient of variation of 1.5%. Serum total cholesterol and triglycerides (TAG) concentrations were measured enzymatically with Roche kits and calibrators on a Cobas Mira analyzer (Roche Diagnostics, Manheim, Germany), as was plasma glucose (Roche Hexokinase Cat. No. 11447513216). Coefficients of variation were 2.8% for total-cholesterol, 4.4% for TAG and 0.5% for plasma glucose. HDL-cholesterol (HDL C) was measured in the supernatant after precipitation of apolipoprotein B containing lipoproteins with phosphotungstate/magnesium chloride solution [22] with a coefficient of variation of 3.6%. LDL-cholesterol was calculated using the Friedwald equation [23]. High sensitivity C-reactive protein (HS-CRP) was measured by latex enhanced immunoturbidimetric method (Roche CRP(Latex) HS Cat. No. 11972855 216) with a coefficient of variation of 4.3%.

Basal metabolic rate was calculated using Harris-Benedict equations [24]. Insulin sensitivity was estimated by the homeostatic model assessment (HOMA-IR 2) using the HOMA-IR 2 calculator [25] and by the McAuley index [26]. A McAuley index value ≤ 6.3 was chosen as a cut-off to define insulin resistance. Presence of the metabolic syndrome (MS) was assessed for each subject based on International Diabetes Federation (IDF) cutoffs [27].

The sample size (n = 35 per group) was determined on the basis of the number of participants required to detect a 1.4 kg (1.8 kg SD) difference in weight loss with 90% power at a level of significance of 0.05. Statistical analysis was performed using the STATA statistical software package 9.0 (Stata, College Station, TX). Data were checked for normality and presented as mean (SD) if normally distributed. Fasting insulin and glucose concentrations were not normally distributed and were thus log-transformed and geometric means (min, max values) are presented. The effect of treatment was analyzed by analysis of covariance with baseline values as a covariate. As this was a proof-of-concept trial data were analyzed on a "per protocol" basis and end of study data were not sought from those who elected to drop out of the study. This is consistent with studies of similar design and duration [13,14,16,18,19,28,29] with which this study is compared.

## Results

Participants were well matched for baseline characteristics (Table 2). Only 6 participants did not meet the IDF criteria for abdominal obesity (WC ≥ 80 cm) at baseline. Over 50% of participants in both groups had elevated total cholesterol concentrations and suboptimal HDL cholesterol concentrations, 32% had blood pressure greater than 130/85 or were taking blood pressure lowering medications. Although only 24% of participants met the IDF criteria for metabolic syndrome 72% had at least 2 risk defining factors.

**Table 2:** Baseline demographic and clinical details for all participants randomized to the high protein (HP) or high fiber (HFib) diets

	HP	HFib
*n*	42	41
Age (years)^a^	40.5 (12.7)	43.4 (9.6)
BMI (kg/m^2^)^a^	33.7 (4.9)	34.2 (4.8)
Weight (kg)^a^	91.5 (15.8)	92.9 (15.3)
Systolic blood pressure (mm Hg)^a^	126 (16)	124 (14)
Diastolic blood pressure (mm Hg)^a^	80 (10)	80 (10)
Triglycerides (mmol/L)^a^	1.47 (0.76)	1.42 (0.62)
HDL Cholesterol (mmol/L)^a^	1.24 (0.27)	1.28 (0.25)
Fasting plasma glucose (mmol/L)^b^	5.0 (3.8, 12.6)	4.9 (3.9, 6.1)
Insulin resistant^c^	9 (21)	8 (20)
Metabolic syndrome^d^	8 (19)	8 (20)

^a ^mean (SD), ^b ^geometric mean (min, max), all other values are n (%); ^c ^McAuley Index ≤ 6.3 Gffm/I; ^d ^International Diabetes Federation definition

Three-day diet records for both baseline and week 8 were returned by 33 and 36 participants in HP and HFib respectively. Reported intakes of total energy and macronutrients at baseline and at week 8 and adjusted differences between the groups are given in Table 3. The data suggest a remarkable degree of compliance with the dietary advice with regard to macronutrient composition. Energy reduction was significantly greater on HFib than on HP. Particularly notable was the difference between the groups with greater intakes of protein and fat in HP and carbohydrate and dietary fiber on HFib. Despite energy reduction of 1664 kJ/d (95% CI: 792, 2536 kJ/d) there was an overall increase in total protein intake of 20 g/d (95% CI: 10, 30 g/d) on HP. This was achieved by increased intake of animal protein (23 g/d [95% CI: 14, 32 g/d]) whereas protein from vegetable sources decreased (-5 g/d [95% CI: 0, 9 g/d]).

**Table 3:** Comparison of changes in dietary intakes^a^

	Baseline mean (SD)	Week 8 mean (SD)	Difference (95% CI)^b^	*P*^c^
Energy (KJ)				
HP	8123 (2153)	6509 (1351)		
HFib	8486 (2000)	5976 (925)	580 (9, 1150)	0.047
Protein (%)				
HP	18 (5)	28 (5)		
HFib	18 (4)	22 (3)	5.6 (3.7, 7.5)	< 0.001
Protein (g)				
HP	82 (22)	104 (16)		
HFib	86 (18)	76 (11)	28 (21, 35)	< 0.001
Animal protein (g)				
HP	53 (19)	76 (14)		
HFib	54 (16)	56 (14)	21 (14, 28)	< 0.001
Fat (%)				
HP	33 (8)	29 (5)		
HFib	31 (7)	23 (6)	5.8 (2.9, 8.8)	< 0.001
Saturated fat (%)				
HP	13 (3)	9 (4)		
HFib	13 (4)	6 (3)	3.0 (1.5, 4.7)	0.001
CHO (%)				
HP	45 (6)	40 (6)		
HFib	47 (7)	51 (6)	-11.1 (-14.2, -8.1)	< 0.001
Dietary fiber (g/day)				
HP	25 (10)	24 (8)		
HFib	26 (7)	39 (11)	-14.2 (-18.9, -9.5)	< 0.001
Soluble dietary fiber (g/day)				
HP	12 (5)	10 (3)		
HFib	12 (3)	16 (5)	-5.2 (-7.2, -3.2)	< 0.001
Insoluble dietary fiber (g/day)				
HP	13 (5)	14 (5)		
HFib	14 (5)	23 (7)	-8.9 (-12.0, -5.9)	< 0.001
Sodium (mg/d)				
HP	2479 (909)	2049 (677)		
HFib	2745 (840)	2125 (498)	-15 (-264, 294)	0.912
Alcohol (g)				
HP	6 (9)	3 (5)		
HFib	5 (9)	2 (5)	0.4 (-1.6, 2.4)	0.693

^a ^High protein diet (HP) n = 33 records, and high fiber diet (HFib), n = 36 records;

^b ^difference between HP and HFib diets estimated by ANCOVA with adjustment for baseline value;

^c ^p-value for the difference between the HP and HFib diets

Participants on the HFib diet reported less hunger (p = 0.036) and less pre-occupation with thoughts of food (p = 0.037) than participants on HP. There were no other differences in measures associated with appetite and satiety. There was no evidence of differences between the groups with regard to measures of self-perceived adherence to the diet, dietary compliance, commitment to dietary change, commitment to weight-loss, likelihood of continuing with the diet plan and health during the dietary intervention at the end of the study.

Both diets were associated with appreciable reductions in total body weight, BMI, fat mass with a significantly greater reduction on HP (Additional File 1). There was little change in lean mass on either diet. Reductions in truncal fat and waist circumference were achieved with both diets but there was no evidence of a difference between the two diets.

Blood pressure levels decreased on both diets but the decrease was not statistically significant on HFib. There was statistically significant greater decrease in DBP on HP than on HFib, but not for SBP (Additional File 2). The reduction in DBP was not influenced by the change in weight. However the change in weight was a statistically significant predictor for the reduction in SBP (p = 0.026) and attenuated the effect of diet (-2.3 mm Hg; 95% CI - 5.8, 1.2 mm Hg; p = 0.197). There were reductions in fasting plasma glucose, insulin, total and LDL cholesterol, triglycerides and HS-CRP and increased insulin sensitivity on both diets but there was no significant effect of diet (Additional File 1). HDL-cholesterol was slightly reduced for both diets but there was no difference between diets (Additional File 2).

## Discussion

Our findings clearly show that both the high protein (HP) and the relatively high carbohydrate, high fiber (HFib) diets were associated with appreciable reductions in total body fat, waist circumference, truncal fat, blood pressure, fasting plasma glucose, total and LDL cholesterol and triglyceride, insulin and an increase in insulin sensitivity. These favorable changes occurred without any loss of lean body mass. However, given the aim of this study, the most important findings relate to the comparisons of the magnitude of the benefits observed with the two dietary prescriptions, the HP diet being associated with a significantly greater reduction in adiposity and diastolic blood pressure when compared with HFib. Improvements in systolic blood pressure, triglyceride concentrations and insulin sensitivity measured by the McAuley Index [26] also tended to be more marked on HP than HFib but these differences did not achieve conventional levels of statistical significance.

Previous studies involving comparisons of weight loss on low fat, high protein (LFHP) and LFHC diets fall into two categories: relatively long term comparisons which provide an indication of what might be achieved in practice using current approaches to implementing weight loss regimes, and shorter term studies which should, at least in theory, provide proof of concept. In a systematic review of 13 randomized controlled weight-loss studies lasting at least six months greater weight loss was observed on high protein diets compared with LFHC diets after 6 and 12 months (- 4 kg and -1 kg respectively) [30]. However there was considerable heterogeneity amongst the studies with the largest of the studies [31] finding no difference between the high protein Zone diet and two conventional LFHC diets. Moreover a recent large population study by Sacks et al. that compared weight-loss diets varying only in the proportions of fat, protein and carbohydrate showed each diet to be equally successful in facilitating and maintaining weight loss over a two-year follow-up period [32]. High rates of attrition and difficulties in assessing long-term compliance make it difficult to conclude with any certainty that high protein diets are indeed superior to LFHC diets in longer-term studies involving free-living individuals. Thus shorter-term "proof of concept" comparisons are required to resolve these issues.

Such studies have generally found comparable, rather than greater, reductions in weight and body fat when comparing isocaloric, energy restricted LFHP diets with LFHC diets in interventions lasting 12 - 16 weeks [11,13,14,18,19,33-35]. However subgroup analyses of some of these studies suggested greater reduction in weight and body fat on LFHP in women with raised triglycerides [18] and obese women with diabetes [36]. Two studies, one of which compared a very high protein (45% TE) diet with a LFHC diet over 4 weeks [17] and the other a 12-wk study in which the high protein diet included an initial 2-week high fat, high protein phase [29], have reported significantly greater weight and fat loss on high protein compared LFHC diets. The differences in experimental design, macronutrient distribution and nature of carbohydrate preclude definitive conclusions from the previously published trials as to whether, even in the short term, relatively high protein intakes confer weight loss and metabolic benefits over relatively high carbohydrate diets rich in dietary fiber derived principally from wholegrains, vegetables and fruits. Our findings suggest that this is indeed the case, at least over a period of 8-wks.

In dietary intervention studies, ranging from 4 weeks to 12 months, participants on ad-libitum high protein diets have reported lower energy consumption than those on other diets and achieved greater weight loss [12,37,38]. Thus high protein diets may facilitate weight loss because subjects spontaneously consume less energy than subjects consuming comparative diets. Only a limited number of studies have examined this issue in the context of metabolically controlled hypocaloric diets. These studies reported increased satiety on high protein versus standard protein diets but this did not translate into a greater reduction of energy intake or more weight loss [11,35,39]. Participants on the HP diet in our study reported greater hunger, more preoccupation with thoughts of food and higher energy intakes than participants on HFib yet lost more weight. Although self-reported energy intakes are notoriously unreliable it would appear that satiation was not a major factor explaining the greater weight loss on HP. The fact that dietary fiber is also associated with increased satiety and fullness [40] may explain these observations.

Weight loss diets have often been associated with some loss of lean body mass in addition to the reduction of fat mass. High protein diets have been associated with retention of lean body mass (LBM) when compared with high carbohydrate diets [16,19,33,41] even when there has been no difference in change in total body weight. A meta-regression analysis of weight loss studies comparing low carbohydrate high protein diets with LFHC diets suggested that protein intakes greater than 1.05 g/kg/d were associated with greater retention of fat free mass compared with lower protein intakes [41]. Thus our findings confirm, once again, the potential of high protein diets to facilitate retention of LBM although the HP diet provided no additional benefit compared with the HFib diet.

Weight loss has been clearly associated with a decrease in blood pressure. A meta-analysis of studies lasting more than 8 weeks examining the effect of weight loss on blood pressure estimated that a 1 kg reduction in weight was associated with reductions of 1.05 mm Hg reduction for SBP and 0.92 mm Hg for DBP [42]. However weight loss explained little of the difference in blood pressure observed on our diets in our statistical models, thus suggesting that there was a specific blood pressure-lowering effect due to protein. Protein intake, from derived primarily from both plant and animal sources, has been associated with lower blood pressure in other dietary intervention studies in the absence of weight loss [35,43-45].

Earlier studies suggesting deleterious effects of high carbohydrate diets relative to high-protein diets may have resulted from the nature of carbohydrate and dietary fiber consumed [15,46]. Participants on HFib in our study chose appropriate carbohydrates and achieved high fiber intakes, and consequently achieved favourable changes in lipids and insulin sensitivity that were comparable with improvements on HP.

Epidemiological studies have suggested that high meat and animal protein intakes are associated with increased risk of insulin resistance syndrome [47] and diabetes [48,49]. Consequently there has been interest in diets emphasizing plant protein as a means to increase overall protein intakes and potentially achieve even greater metabolic benefits than high protein diets emphasizing animal protein [35,43]. Our findings, however, show no evidence of deleterious effects on metabolic risk factors of a substantial increase in animal protein on the HP diet.

The fact that our participants were relatively healthy, despite being overweight, may have limited our ability to show the potential of the two diets to modify metabolic risk factors. There is evidence to suggest that macronutrient composition may have a greater influence on metabolic responses in individuals with certain metabolic risk factors compared to those without such risk factors [50,51]. While this evidence suggests that diets relatively higher in protein may be more beneficial for high-risk individuals than high carbohydrate diets, direct comparisons between high protein and high carbohydrates diets emphasizing high fiber, minimally processed foods have not been made. Indeed other studies suggest that high fiber diets improve insulin sensitivity and other cardiovascular risk factors in individuals with hyperinsulinaemia and diabetes in comparison with diets high in refined carbohydrates [52,53]. Thus the generalisability of our research to very high risk individuals, such as those with diabetes, is uncertain.

The relatively short duration of our study may also be considered to be a limitation. Longer-term studies indicate that short-term benefits associated with reduced carbohydrate intakes are not sustained over the longer-term but this is largely explained by subjects reverting to their usual dietary patterns [30,32,54]. This dietary intervention trial was intended to be a "proof-of-concept" study with the clear objective being to compare the potential of weight reducing diets differing in macronutrient composition to influence measures of adiposity and metabolic risk factors. In this respect the remarkable extent to which participants complied with the dietary prescriptions and the use of DXA as a relatively sensitive measure for assessing adiposity are major strengths. Clearly these findings relate most directly to overweight and obese women, but there is only limited evidence to suggest that men might respond differently [36].

## Conclusions

We believe that we have demonstrated modest overall benefit when a relatively high protein weight-reducing diet has been compared with a high-fiber diet. However our findings suggest that considerable benefit may also accrue from a diet that is rich in wholegrain cereals, legumes, intact fruits and vegetables and low in saturated fat. Earlier studies suggesting deleterious effects of high carbohydrate diets relative to high protein diets may have resulted from the nature of carbohydrate and dietary fiber consumed [15,46]. On the basis of these findings it seems reasonable to conclude that that while a high protein diet might be the preferred prescription for weight reduction, a relatively high carbohydrate diet offers an alternative for those who, because of cost or dietary preferences, choose not to increase protein intake. Given that the present study was a relatively short-term proof-of-concept study and longer-term studies have generated equivocal results, novel approaches are needed to examine ways of encouraging long-term adherence to dietary advice before conclusions can be drawn with regard to the long-term importance of macronutrient composition.

## Competing interests

JM and LAT have received funding from Fonterra Co-operative Group Ltd which has facilitated this and other projects. Fonterra have had no involvement in the design, analysis, interpretation or reporting of the data. None of the authors had a conflict of interest.

## Authors' contributions

LAT was the main author of manuscript and participated in the study design, execution and data collection, and carried out the statistical analyses. MTL contributed to the execution of the study, data collection, analysis and interpretation. SMW supervised the statistical analyses and assisted with the preparation of the manuscript. RCB and JM were doctoral supervisors of LAT and participated in the study conception, design, interpretation and preparation of the manuscript. All authors read and approved the final manuscript.

## Supplementary Material

Additional file 1 **Table S1: Comparison of body composition outcomes for all participants completing the study**. Results table that does not fit to a single portrait page width. Click here for file

Additional file 2 **Table S2: Comparison of biochemical measures for for all participants completing the study**. Results table that does not fit to a single portrait page width. Click here for file
