# Supplementary material for: Comparison of high protein and high fiber weight-loss diets in women with risk factors for the metabolic syndrome: a randomized trial
Source: Nutr J. 2011 Apr 28;10:40. doi: 10.1186/1475-2891-10-40 (PMC3105953; doi:10.1186/1475-2891-10-40)
Supplement: Additional file 1 — Table S1: Comparison of body composition outcomes for all participants completing the study. Results table that does not fit to a single portrait page width. [file 1475-2891-10-40-S1.PDF]

**Table S1: Comparison of body composition outcomes for all participants completing the study.**

| Variable | *n* | Baseline Mean (SD) | Week 8 Mean (SD) | Change (95% CI)[a] | Difference between diets[b] | *P*[c] |
|---|---|---|---|---|---|---|
| Weight (kg) | | | | | | |
| HP | 37 | 89.8 (14.9) | 85.3 (14.7) | - 4.5 (-3.7, -5.4) | -1.3 (-2.5, -0.1) | 0.039 |
| HFib | 37 | 91.8 (14.5) | 88.5 (14.9) | -3.3 (-2.4, -4.2) | | |
| BMI (kg/m$^2$) | | | | | | |
| HP | 37 | 32.9 (4.6) | 31.3 (4.5) | -1.7 (-1.4, -2.0) | -0.5 (-0.9, -0.1) | 0.020 |
| HFib | 37 | 33.6 (4.4) | 32.5 (4.7) | -1.2 (-0.9, -1.5) | | |
| Total body fat (kg) | | | | | | |
| HP | 34 | 38.2 (7.8) | 34.2 (8.5) | -4.0 (-4.6, -3.4) | -1.3 (-2.4, -0.1) | 0.029 |
| HFib | 36 | 41.8 (10.1) | 39.3 (10.7) | -2.5 (-3.5, -1.6) | | |
| Total body fat (%) | | | | | | |
| HP | 34 | 44.2 (4.4) | 41.5 (5.5) | -2.7 (-3.2, -2.1) | -0.9 (-1.9, -0.3) | 0.049 |
| HFib | 36 | 46.1 (5.5) | 44.5 (6.6) | -1.5 (-2.3, -0.7) | | |
| Total lean mass (kg) | | | | | | |
| HP | 34 | 45.0 (5.0) | 44.8 (5.1) | -0.2 (-0.6, 0.2) | 0.3 (-0.4, 0.9) | 0.414 |
| HFib | 36 | 45.3 (4.7) | 44.9 (4.9) | -0.4 (-0.9, 0.04) | | |
| Truncal body fat (kg) | | | | | | |
| HP | 34 | 19.3 (4.6) | 16.7 (5.1) | -2.6 (-3.1, -2.2) | -0.6 (-1.4, 0.1) | 0.103 |
| HFib | 36 | 21.5 (5.5) | 19.6 (5.9) | -1.8 (-2.5, -1.3) | | |
| Waist (cm) | | | | | | |
| HP | 37 | 93.5 (10.8) | 88.3 (10.4) | -5.4 (-6.3, -4.5) | -0.9, (-2.4, 0.5) | 0.183 |
| HFib | 37 | 97.2 (12.0) | 92.5 (11.4) | -4.7 (-3.6, -5.8) | | |
| SBP[d] (mm Hg) | | | | | | |
| HP | 37 | 126 (16) | 121 (15) | -5.4 (-8.4, -2.4) | -3.1 (-6.8, 0.6) | 0.095 |
| HFib | 37 | 124 (14) | 122 (13) | -1.7 (-4.4, 1.0) | | |
| DBP[e] (mm Hg) | | | | | | |
| HP | 37 | 80 (10) | 75 (8) | -4.7 (-6.7, -2.8) | -3.7 (-6.2, -1.1) | 0.005 |
| HFib | 37 | 80 (10) | 79 (10) | -0.9 (-3.1, 1.2) | | |

[a] Change estimated by paired t-test; [b] difference between HP and HFib diets estimated by ANCOVA with adjustment for baseline value; [c] p-value for the difference between the HP and HFib diets, [d]systolic blood pressure, [e]diastolic blood pressure .
