# Supplementary material for: Comparison of high protein and high fiber weight-loss diets in women with risk factors for the metabolic syndrome: a randomized trial
Source: Nutr J. 2011 Apr 28;10:40. doi: 10.1186/1475-2891-10-40 (PMC3105953; doi:10.1186/1475-2891-10-40)
Supplement: Additional file 2 — Table S2: Comparison of biochemical measures for for all participants completing the study. Results table that does not fit to a single portrait page width. [file 1475-2891-10-40-S2.DOCX]

**Table S2: Comparison of biochemical measures for for all participants completing the study.**

| Variable | | *n* | | Baseline^a^ | | Week 8 | | Change^b^ | | Difference between diets^c^ | | *P*^d^ | |
| --- | --- | --- | --- | --- | --- | --- | --- | --- | --- | --- | --- | --- | --- |
| Fasting plasma glucose (mmol/L)^e^  HP  HFib | | 37  37 | | 5.0 (3.8, 12.6)  4.9 (3.9, 6.1) | | 4.9 (4.1, 11.9)  4.8 (3.9, 5.8) | | -1.9% (-3.9%, 0%)  -2.6% (-4.9%, -0. 4%) | | 0.9% (-1.9%, 3.7%) | | 0.527 | |
| Fasting serum insulin (μIU/ml)^e^  HP  HFib | | 37  37 | | 9.6 (4.8, 31.8)  9.9 (3.9, 38.4) | | 8.2 (3.7, 68.9)  8.8 (1.7, 43.1) | | -17.9% (-33.3%, -4.3%)  -12.6% (-29.1%, 1.7%) | | -9.2% (-28.7%, 15.7%) | | 0.430 | |
| Total Cholesterol (mmol/L)  HP  HFib | | 37  37 | | 5.3 (1.1)  5.1 (0.9) | | 4.7 (0.9)  4.7 (0.7) | | -0.62 (-0.80, -0.45)  -0.46 (-0.66, -0.27) | | -0.11 (-0.33, 0.11) | | 0.330 | |
| LDL Cholesterol (mmol/L)  HP  HFib | | 37  37 | | 3.3 (0.9)  3.2 (0.7) | | 3.0 (0.8)  2.9 (0.6) | | -0.37 (-0.51, -0.24)  -0.31 (-0.47, -0.15) | | -0.02 (-0.21, 0.16) | | 0.791 | |
| HDL Cholesterol (mmol/L)  HP  HFib | | 37  37 | | 1.3 (0.3)  1.3 (0.2) | | 1.2 (0.3)  1.2 (0.3) | | -0.10 (-0.14, -0.06)  -0.07 (-0.13, -0.01) | | -0.04 (-0.11, 0.03) | | 0.280 | |
| Triglycerides (mmol/L)  HP  HFib | | 37  37 | | 1.5 (0.8)  1.4 (0.6) | | 1.2 (0.5)  1.2 (0.5) | | -0.31 (-0.43, -0.19)  -0.18 (-0.29, -0.08) | | -0.09 (-0.20, 0.02) | | 0.111 | |
| HOMA2 insulin sensitivity (%)  HP  HFib | | 37  37 | | 70.1 (22.1, 139.7)  68.5 (18.3, 177.9) | | 83.2 (10.9, 191.6)  76.6 (16.2, 254.7) | | 15.7% (4.9%, 25.3%)  10.6% (-1.5%, 21.2%) | | 6.1% (-10.8, 26.1%) | | 0.501 | |
| McAuley Index  HP  HFib | | 37  37 | | 6.9 (1.6)  6.9 (1.4) | | 7.7 (1.9)  7.4 (1.8) | | 0.8 (0.5, 1.1)  0.5 (0.2, 0.9) | | 0.3 (-0.2, 0.8) | | 0.263 | |

^a^ Mean (95% confidence interval); ^b^ change estimated by paired t-test; ^c^ difference between HP and HFib diets estimated by ANCOVA with adjustment for baseline value; ^d^ p-value for the difference between the HP and HFib diets; ^e^ geometric mean (min, max)
